# Cryo-EM structure of the photosynthetic RC-LH1-PufX supercomplex at 2.8-Å resolution

**DOI:** 10.1126/sciadv.abf8864

**Published:** 2021-06-18

**Authors:** Laura Bracun, Atsushi Yamagata, Bern M. Christianson, Tohru Terada, Daniel P. Canniffe, Mikako Shirouzu, Lu-Ning Liu

**Affiliations:** 1 Institute of Systems, Molecular and Integrative Biology, University of Liverpool, Liverpool L69 7ZB, UK.; 2 Laboratory for Protein Functional and Structural Biology, RIKEN Center for Biosystems Dynamics Research, 1-7-22 Suehiro-cho, Tsurumi-ku, Yokohama, Kanagawa 230-0045, Japan.; 3 Department of Biotechnology, Graduate School of Agricultural and Life Sciences, University of Tokyo, 1-1-1 Yayoi, Bunkyo-ku, Tokyo 113-8657, Japan.; 4 College of Marine Life Sciences and Frontiers Science Center for Deep Ocean Multispheres and Earth System, Ocean University of China, Qingdao 266003, China.

## Abstract

The reaction center (RC)−light-harvesting complex 1 (LH1) supercomplex plays a pivotal role in bacterial photosynthesis. Many RC-LH1 complexes integrate an additional protein PufX that is key for bacterial growth and photosynthetic competence. Here, we present a cryo–electron microscopy structure of the RC-LH1-PufX supercomplex from *Rhodobacter veldkampii* at 2.8-Å resolution. The RC-LH1-PufX monomer contains an LH ring of 15 αβ-polypeptides with a 30-Å gap formed by PufX. PufX acts as a molecular “cross brace” to reinforce the RC-LH1 structure. The unusual PufX-mediated large opening in the LH1 ring and defined arrangement of proteins and cofactors provide the molecular basis for the assembly of a robust RC-LH1-PufX supercomplex and efficient quinone transport and electron transfer. These architectural features represent the natural strategies for anoxygenic photosynthesis and environmental adaptation.

## Teaser

Atomic structure of the first RC-LH1-PufX supercomplex provides new insights into the assembly and function of photosynthetic systems.

## INTRODUCTION

Photosynthesis, performed by plants, algae, and many bacteria, is one of the most important biological processes on Earth as it harnesses solar energy to provide energy, oxygen, and food for life (*1*, *2*). In purple bacteria, photosynthesis requires close connectivity between several membrane protein complexes: the peripheral light-harvesting complex 2 (LH2), the central LH1, the photochemical reaction center (RC), the proton translocating cytochrome (Cyt) *bc*_1_ complex, and adenosine 5′-triphosphate (ATP) synthase (*3*, *4*). Typically, the RC and LH1 form a photosynthetic core supercomplex that is central to bacterial photosynthesis (*5*). The photoinduced charge separation results in the reduction of quinone to quinol in the RC, which is then translocated across LH1 and diffuses through the membrane to Cyt *bc*_1_, where a proton-motive force is generated to trigger ATP synthesis. Cyt *bc*_1_ reoxidizes the quinol, and an electron is shuttled via the soluble cytochrome *c*_2_ back to the RC.

In the past few years, several RC-LH1 complexes from different purple photosynthetic bacteria have been resolved, exhibiting the distinctive molecular architectures of the photosynthetic supercomplexes. The RC-LH1 complexes from *Thermochromatium* (*Tch*.) *tepidum* and *Thiorhodovibrio* (*Trv*.) strain 970 contain a closed LH1 ring of 16 αβ-heterodimers enclosing the RC with an extra periplasmic tetrahaem cytochrome subunit (4Hcyt) (*6*–*8*). In the bacteriochlorophyll (BChl) *b*–producing bacterium *Blastochloris* (*Blc.*) *viridis*, the 4Hcyt-containing RC is encircled by an LH1 ring that consists of 16 αβγ-heterotrimers and one αβ-heterodimer, and the missing γ-polypeptide creates a small gap in the LH1 ring (*9*, *10*). In *Rhodopseudomonas* (*Rps.*) *palustris*, the RC is encircled by an open LH1 ring consisting of 14 αβ-heterodimers and protein W (*11*, *12*), whereas the LH1 ring of *Roseiflexus* (*Rfl*.) *castenholzii* is interrupted by the transmembrane helix of Cyt *c* and subunit X (*13*). While it has been proposed that quinone/quinol can diffuse across the LH1 ring through specific channels between LH1 αβ-heterodimers (*6*–*8*), the gap in the LH1 ring potentially offers unique routes to facilitate quinone/quinol exchange between the RC and Cyt *bc*_1_.

The RC-LH1 core complex of most *Rhodobacter* species contains an additional transmembrane polypeptide PufX of ~80 amino acids, which is involved in determining the quinone diffusion and dimerization of the RC-LH1 complex (*14*–*17*). An exception to this arrangement is *Rhodobacter* (*Rba*.) *veldkampii* (*18*), in which the RC-LH1 core complexes form only monomers in the presence of PufX, as characterized by atomic force microscopy (AFM) (*5*) and low-resolution cryo–electron microscopy (cryo-EM) (*19*, *20*). However, because of the limited resolution, the molecular basis underlying the assembly and arrangement of proteins and cofactors within this specific RC-LH1-PufX supercomplex to ensure efficient electron transfer remains unclear. Here, we report a cryo-EM structure of this RC-LH1-PufX photosynthetic complex from *Rba*. *veldkampii* at 2.8-Å resolution. Analysis of the supercomplex structure reveals how PufX functions as a molecular cross brace to interact with the RC, LH1, and cofactors, and thereby plays roles in determining the overall architecture and quinone transport pathways of the RC-LH1-PufX complex.

## RESULTS AND DISCUSSION

### Overall structure

**Fig. 1:**
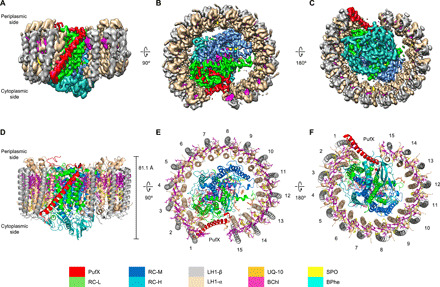
Cryo-EM structure of the RC-LH1-PufX core complex from *Rba. veldkampii*. (**A** to **C**) Color-coded electron density map in three different views. Color scheme is presented in the legend at the bottom as follows: LH1-α, wheat; LH1-β, gray; PufX, red; RC-L, green; RC-M, marine; RC-H, teal; BChls, purple; BPhes, cyan; carotenoids, yellow; and quinones, orange. (A) Side view of the RC-LH1-PufX complex in the membrane plane. (B) Top view of the RC-LH1-PufX complex from the periplasmic side. (C) Bottom view of the RC-LH1-PufX complex from the cytoplasmic side. (**D** to **F**) Architectural model of the RC-LH1-PufX complex in three different views corresponding to (A to C). The height of the RC-LH1-PufX complex perpendicular to the membrane bilayer is shown in (D). The LH1 subunits are numbered in (E) and (F).

The intact RC-LH1-PufX core complexes were isolated from photoheterotrophically grown *Rba. veldkampii* cells (*21*). The *Rba. veldkampii* RC-LH1-PufX complexes are present exclusively as monomers with a single conformation, as displayed by reference-free two-dimensional (2D) and subsequent 3D classifications (fig. S1). From a total of 1,168,396 particles, 184,921 good particles were used for final 3D reconstruction. Cryo-EM single-particle analysis determined the structure of the RC-LH1-PufX core complex at 2.84-Å resolution (fig. S2). The cryo-EM map clearly defined most of the amino acid side chains (Fig. 1, A to C, and fig. S3) and enabled the generation and refinement of the atomic model of the RC-LH1-PufX complex (Fig. 1, D to F, and table S1).

The overall structure of the *Rba. veldkampii* RC-LH1-PufX complex is composed of H, L, and M subunits for the RC, α_15_β_15_ subunits for LH1, the transmembrane peptide PufX, and 62 cofactors (Fig. 1 and table S2). The total molecular mass of RC-LH1-PufX is ∼285 kDa. The height of the core complex from the C terminus of PufX on the periplasmic side to the bottom of the H subunit on the cytoplasmic side is 81.1 Å (Fig. 1D). The RC contains three protein subunits (H, L, and M), four BChls a, three bacteriopheophytins (BPhes), one carotenoid, one Fe^3+^ ion, and six ubiquinone molecules tentatively assigned as ubiquinone-10 (UQ-10) due to its high content in previously studied *Rhodobacter* species (*22*). The RC is surrounded by the LH1 ring composed of 15 pairs of transmembrane helices of α- and β-apoproteins (Fig. 1, E and F), in agreement with the structures determined by AFM and low-resolution cryo-EM (*19*, *20*). The overall LH1 ring forms a slightly elliptical architecture, with the lengths of 113.7 and 107.6 Å for the major and minor axes, respectively (fig. S4).

### Interactions between PufX and RC-LH1

**Fig. 2:**
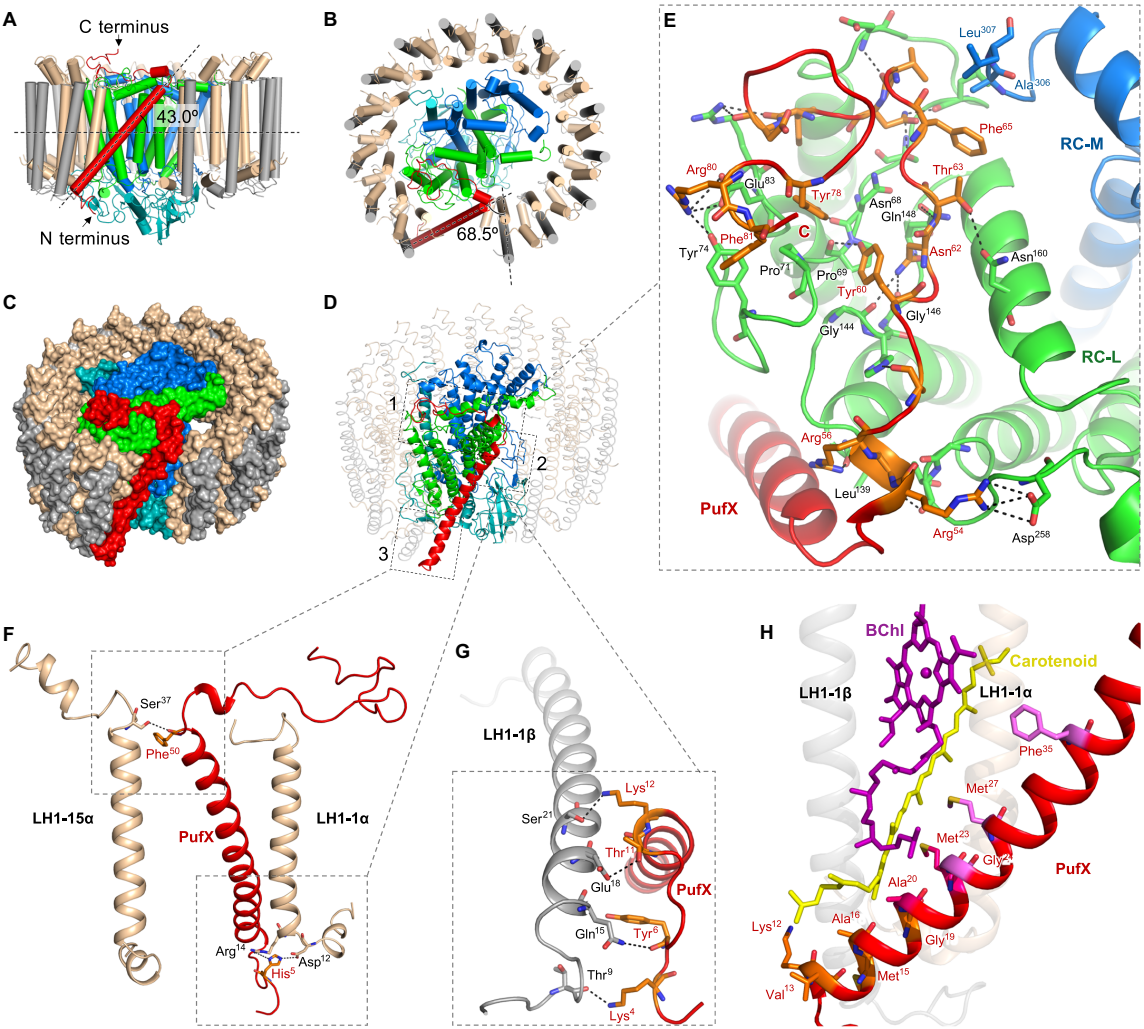
The PufX structure and interactions within the RC-LH1-PufX complex. (**A** and **B**) Schematic model of the RC-LH1-PufX complex represented by cylinders. PufX (red cylinder) exhibits a tilt angle of 43° to the membrane plane (A) and an angle of 68.5° to the orientation of LH1-15αβ heterodimer (B). (**C**) Color-coded protein surface representation of the RC-LH1-PufX complex. Color scheme is as depicted in Fig. 1. (**D**) Cartoon representation of the RC-LH1-PufX complex. Interaction sites between PufX and the RC-LH1 assembly are boxed. (**E**) The interaction network between PufX and RC-L subunit [box 1 in (D)]. Selected PufX residues that interact with RC-L are shown in orange. Selected RC-L residues that interact with PufX are colored green and shown in sticks. All the interacting residues involved in the association between PufX and the RC-L subunit are listed in table S3. (**F**) The interactions between PufX and LH1-1α and LH1-15α on both sides of the gap in the LH1 ring [boxes 2 and 3 in (D)]. Interacting residues are shown in sticks. (**G**) The interactions between PufX and LH1-1β [(box 3 in (D)]. Interacting residues are shown in sticks. (**H**) The interface between PufX and pigments within the LH1-1αβ heterodimer, formed by hydrophobic interactions. Interacting residues are shown in sticks and colored orange if they are interacting with the carotenoid, purple if interacting with BChl, and magenta if interacting with both. PDB ligand ID: BChl, BCL; spheroidene, SPO; and UQ-10, U10.

A unique feature of the *Rba. veldkampii* RC-LH1-PufX complex is a marked gap (30 Å in distance) with the LH1 ring interrupted by PufX (Fig. 2 and fig. S4). The PufX peptide is composed of a central bent transmembrane helix (from Thr^11^ to Gln^49^), a short N-terminal tail and a C-terminal loop. The transmembrane helix of PufX has a tilt angle of 43.0° to the membrane plane and an angle of 68.5° to the orientation of the 15th LH1 αβ-heterodimer (Fig. 2, A and B), instead of being parallel to the LH1 αβ-peptides (*20*). The superposition with the nuclear magnetic resonance structure of isolated PufX from *Rba. sphaeroides* [Protein Data Bank (PDB) ID: 2DW3] (*23*) revealed that the transmembrane helices of the two structures, which are conserved among the PufX-containing species, are well fitted, whereas the extended N- and C-terminal regions show flexible conformations (fig. S5).

The N-terminal tail of PufX is exposed on the cytoplasmic surface of the photosynthetic membrane, close to the N terminus of LH1-1β (Fig. 2, C and D, box 3). The C-terminal domain of PufX is exposed on the periplasmic side of the RC-LH1-PufX complex (Fig. 2, C and D, box 1). The C-terminal loop is kinked by the interactions between two arginine residues (Arg^54^ and Arg^56^) of PufX with Asp^258^ and Leu^139^ of the RC-L subunit, respectively, and is stabilized by extensive hydrophilic and hydrophobic interactions with the RC-L subunit (Fig. 2E, fig. S6, and table S3). Specifically, PufX Tyr^60^ and Tyr^78^ are stabilized by hydrogen bonds with the main-chain carbonyls of L-Pro^69^ and L-Asn^68^ (Fig. 2E). The Tyr^60^ and Tyr^78^ residues of PufX are also packed toward the L subunit through hydrophobic interactions with L-Pro^69^ and L-Pro^71^. The side chain of PufX Asn^62^ forms hydrogen bonds with L-Gly^144^, L-Gly^146^, and L-Gln^148^. PufX Thr^63^ forms a hydrogen bond with L-Asn^160^. Near the C terminus, the side chain of PufX Arg^80^ is hydrogen bonded to L-Tyr^74^ and forms a salt bridge with the side chain of L-Glu^83^. PufX Phe^81^ is exposed to a hydrophobic patch formed by Pro^71^, and Tyr^74^ of the L subunit. Other interactions in this interface are listed in table S3. In addition, the M subunit of the RC partially contributes to the PufX-RC interface as PufX Phe^65^ hydrophobically interacts with M-Ala^306^ and M-Leu^307^ (Fig. 2E).

The PufX-binding sites in the L subunit are largely conserved among purple bacteria (fig. S6), suggesting high compatibility of PufX binding to the RC. This is indicated by the superimposed structures of *Rba. veldkampii* PufX-RC-L and *Rba. sphaeroides* Cyt *c*_2_–RC (PDB ID: 1L9J) (*24*). Moreover, the PufX-RC association does not affect the binding of the water-soluble electron carrier Cyt *c*_2_ to the RC, which occurs at the center of the periplasmic surface of the RC (fig. S7). Therefore, the specific PufX-RC binding does not affect reduction of the oxidized special pair in the *Rhodobacter* genus of purple photosynthetic bacteria. However, the superpositions of the 4Hcyt subunit cause a steric hindrance to the binding of PufX C-terminal loop to the RC (fig. S7), explaining why many purple bacteria (especially those containing PufX) do not have a 4Hcyt subunit and rely on Cyt *c*_2_ or other electron carriers (i.e., Cyt *c*_y_) for electron donation (*10*).

The specific orientation of the transmembrane helix of PufX is mediated by interactions with adjacent LH1 αβ-heterodimers located next to the gap in the LH1 ring. The middle region of PufX forms close contacts with the periplasmic side of the LH1-15α apoprotein (Fig. 2D, box 2). An inter-subunit hydrogen bond between PufX and LH1-15α is formed between α-Ser^37^ and PufX-Phe^50^ at the inner side of the LH1 ring (Fig. 2F and table S4). No direct interactions are formed between PufX and the LH1-15β apoprotein. At the interface between PufX and LH1-1α on the other side of the gap, PufX-His^5^ forms a salt bridge and hydrogen bond with α-Asp^12^ and α-Arg^14^, respectively. The PufX-LH1-1β interface forms hydrogen-bonding networks between Lys^4^, Tyr^6^, Thr^11^, and Lys^12^ residues of PufX and Thr^9^, Gln^15^, Glu^18^, and Ser^21^ of LH1-1β, respectively (Fig. 2G and table S4). The specific molecular architecture suggests the assembly pathway of the RC-LH1-PufX supercomplex. Presumably, the assembly of the photosynthetic core complex initiates with the association of PufX and the RC in the membrane, and then PufX serves as an anchoring site for LH1 α/β-subunits to trigger LH1 assembly and encirclement.

PufX is also involved in stabilizing BChl *a* and carotenoid molecules in the LH1-1 αβ-heterodimer, mainly through hydrophobic interactions (Fig. 2H and table S5). The periplasmic end of the carotenoid is in close proximity to the N-terminal domain of PufX (Lys^12^, 3.5 Å; Val^13^, 5.6 Å; Met^15^, 4.9 Å; Ala^16^, 3.4/3.7 Å; Gly^19^, 4.3 Å; Ala^20^, 3.6 Å; and Met^23^, 3.7 Å). BChl a has close contacts with the hydrophobic residues Ala^20^ (3.5 Å), Met^23^ (4.1 Å), Gly^24^ (3.8 Å), Met^27^ (4.1 Å), and Phe^35^ (5.0 Å). Among them, the Ala^20^ and Met^23^ residues that are highly conserved in the PufX-containing species interact with both BChl a and carotenoid (Fig. 2H, fig. S5, and table S5).

PufX in *Rba. sphaeroides* is crucial for mediating the dimerization of the RC-LH1 core complex (*15*, *16*). The cryo-EM structure of the *Rba. veldkampii* RC is similar to the crystal structure of the *Rba. sphaeroides* RC (PDB ID: 2J8C) (fig. S8), except for their lipid compositions (*25*, *26*). The superimposed structures of two *Rba. veldkampii* RC-LH1-PufX monomers and a *Rba. sphaeroides* RC-LH1-PufX dimer (7.78 Å; PDB ID: 4V9G) (*27*) depicted that two PufX proteins might be located at the center of the RC-LH1 dimer (fig. S8). This implies that the outward-facing N-terminal regions of two PufX peptides may form an interface at the cytoplasmic side, suggesting the importance of the PufX N terminus in the dimerization of *Rba. sphaeroides* RC-LH1-PufX complexes as previously assumed (*28*). A higher-resolution structure of the dimeric RC-LH1-PufX complex is required to provide mechanistic details.

### RC-LH1 interactions

**Fig. 3:**
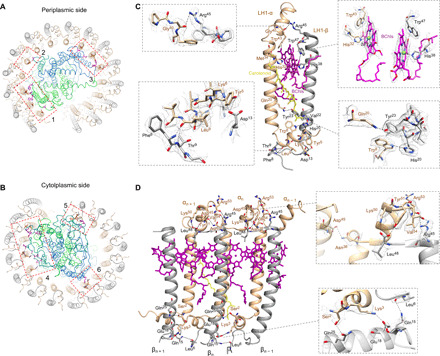
Protein-protein and protein-pigment interactions within the RC-LH1 association. (**A** and **B**) Interaction sites (boxed) between the RC and LH1 at the periplasmic side (A) and the cytoplasmic side (B). (**C**) Interactions within an LH1 subunit. Interacting residues are shown in sticks. Hydrophobic interactions with the carotenoid molecule are shown by yellow dashed lines. (**D**) Intra- and inter-subunit interactions within three LH1 αβ-subunits. Interacting residues are shown in sticks. Details are displayed by zoomed-in views showing residue electron densities and interactions [(C) and (D), boxes]. Distances between interacting residues are shown in table S7.

LH1 generates six binding interfaces with the RC at both the periplasmic and cytoplasmic sides (Fig. 3, A and B). At the periplasmic side, L-Trp^52^, L-Trp^60^, L-Ala^79^, M-Asp^80^, M-Ala^108^, M-Met^111^, and H-Phe^7^ interact with the Ser^37^ residues of neighboring LH1 α-subunits (Fig. 3A, fig. S9, and table S6). At the cytoplasmic side, two arginine residues (Arg^14^ and Arg^15^) of the α-subunit helices have a major role in interacting with the RC subunits (table S6). On the outer side of the LH1 ring, LH1-15β is the only β-subunit that directly interacts with the RC via hydrogen bonds of Ser^7^ and Phe^8^ residues with His^201^ and Lys^199^ of the RC-H subunit, respectively (Fig. 3B, box 5, fig. S9, and table S6). Moreover, the integration of the molecular cross brace PufX leads to a strong association between the RC and LH1. All of these interactions provide the foundation for forming a robust RC-LH1-PufX supercomplex.

### Intra- and inter-subunit interactions within LH1

**Fig. 4:**
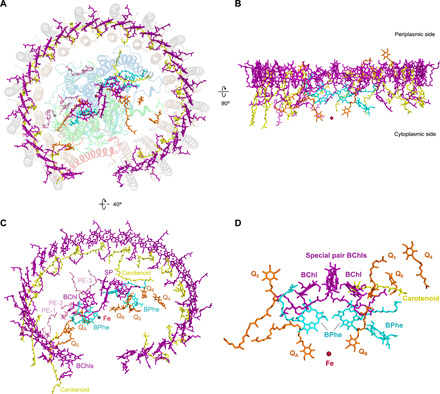
Arrangement of the pigments and cofactors in the *Rba. veldkampii* RC-LH1-PufX complex. (**A**) Periplasmic view of the supramolecular organization of pigments and cofactors in the complex. Protein subunits are depicted in transparent cartoon. (**B**) Side view of the architecture of pigments and cofactors in the membrane plane with the periplasm above and the cytoplasm below. (**C**) Pigments and cofactors viewed from the periplasmic side by tilting 40°. Color scheme: BChls, purple; BPhes, cyan; carotenoid, yellow; quinones, orange; Fe, firebrick; and lipids, pink. (**D**) Arrangement of the pigments, iron, and quinones associated with the RC.

The LH1 ring is formed by the transmembrane helices of the inner α- and outer β-apoproteins, both with the N and C termini located at the cytoplasmic and periplasmic sides, respectively (Fig. 1D). Both N and C termini of the LH1 α-apoprotein form additional helices; the cytoplasmic helix of the LH1 α-apoprotein is perpendicular to the β-apoprotein (Fig. 3, C and D). Each LH1 αβ-heterodimer contains two B880 BChls a at the periplasmic side and one carotenoid molecule, assigned as spheroidene based on the spectral properties (absorption maxima at 446, 471, and 505 nm) (Fig. 3C, fig. S10, and table S2) (*18*, *21*, *29*). The cryo-EM structure does not identify the spheroidene molecule within the 15th LH1 αβ-heterodimer that is next to the LH1 opening. The B880 pigments bound in the same and adjacent LH1 αβ-heterodimers overlap with each other in parallel to form the circular array of pigments (Figs. 3D and 4A). All 15 αβ-heterodimers can be superimposed over the transmembrane region with good agreement (fig. S11). However, the N-terminal domain of the α-apoprotein shows relatively large deviations, especially those located next to the gap (LH1-1 and LH1-15), presumably due to the changes in the local membrane environment resulting from the specific interactions between PufX and LH1 subunits.

Within the individual LH1 αβ-heterodimer, α-Tyr^5^, α-Trp^8^, and α-Leu^9^ form hydrogen bonds with β-Asp^13^, β-His^20^, β-Phe^8^, and β-Thr^9^ (Fig. 3C and table S7). The α-subunit Lys^6^ interacts with β-Asp^13^ via salt bridge at the cytoplasmic side. Hydrogen bonds were also found between α-Gln^20^ and β-Tyr^23^ in the transmembrane region and between α-Gly^40^ and β-Arg^45^ at the periplasmic side. In addition, α-Met^34^ and β-Val^22^ are in close proximity to the ends of the spheroidene molecule (Fig. 3C). Both α-Trp^43^ and β-Trp^47^ are hydrogen bonded to the keto-oxygens of BChl a, and the Mg atoms of BChls *a* are coordinated by their neighboring α-His^32^ and β-His^38^ residues. Collectively, these interactions ensure tight associations of the αβ-apoproteins and pigments within the LH1 αβ-heterodimer assembly.

The cryo-EM structure also reveals intermolecular interactions between LH1 αβ-heterodimers in the *Rba. veldkampii* RC-LH1-PufX complex. Extensive inter-subunit hydrogen bonds between LH1 αβ-heterodimers are formed in the C-terminal regions at the periplasmic side (Fig. 3D and table S7). β_(*n*)_-Leu^48^ is hydrogen bonded to its neighboring α_(*n*)+1_ residues Lys^50^ and Tyr^51^; the β_(*n*)_-Arg^45^ residue, which is highly conserved in purple bacteria, forms hydrogen bonds with α_(*n*) + 1_-Arg^53^ and α_(*n*)+1_-Val^54^. At the cytoplasmic side, α_(*n*)−1_-Ser^2^ is hydrogen bonded to β_(*n*)_-Gln^25^, and α_(*n*)−1_-Lys^3^ forms a interaction with the neighboring β_(*n*)_-Glu^18^ residue via salt bridge. In addition, interactions between adjacent α-subunits are formed between α_(*n*)_-Asn^38^ and α_(*n*)+1_-Asp^45^ at the periplasmic side. Hydrogen bonds are also formed between adjacent β-subunits through the interaction between β_(*n*)_-Leu^6^ and β_(*n*)+1_-Gln^15^ in the N-terminal region at the cytoplasmic side. As a consequence, the LH1 α- and β-subunits form extensive intra- and inter-subunit interactions with their neighboring α- and β-subunits (fig. S12). These interactions sufficiently stabilize the LH1 protein-protein association even without Ca^2+^ that plays roles in the LH1 assembly of RC-LH1 complexes from *Tch. tepidum* (*6*, *7*) and *Trv*. strain 970 (*8*). This provides the structural basis for the formation of a rigid LH1 ring architecture, integration of cofactors within the LH1, and the association of LH1 and the RC.

### Arrangement of cofactors

Thirty B880 BChls *a* and 14 spheroidene molecules are accommodated in the interhelical space of an LH1 αβ-heterodimer and constitute a tightly stacked array in the LH1 ring (Fig. 4, A and B). Equal Mg-Mg distances were found between B880 BChls in individual LH1 αβ-heterodimers (9.53 Å on average) and between BChls of adjacent LH1 αβ-subunits (8.42 Å on average) (fig. S13 and table S8), both within 10 Å, critical for efficient exciton coupling and energy resonance within LH1. The average Mg-Mg intra-subunit distance (9.53 Å) is greater than those of the RC-LH1 complexes from *Tch. tepidum* (8.88 Å) (*7*), *Trv*. strain 970 (9.01 Å) (*8*), and *Blc. viridis* (8.8 Å) (*9*), while it is closer to those of RC-LH1 from *Rfl. castenholzii* (9.5 Å) and LH2 (9.5 Å) (*30*). Coincidently, the Q*_y_* band of closely associated BChls *a* in the *Rba. veldkampii* RC-LH1-PufX core complex is at 884 nm (fig. S10), comparable to that of *Rfl. castenholzii* RC-LH1 (882 nm) (*31*) but less than those of the red-shifted RC-LH1 complexes from *Tch. tepidum* (915 nm), *Trv*. strain 970 (960 nm), and *Blc. viridis* (1008 nm). This suggests the potential correlation between the average intra-subunit Mg-Mg distances within BChl pairs and excitonic coupling of BChls *a* in LH complexes (*9*).

The spheroidene molecules are the major carotenoids in *Rhodobacter* species (*29*). Spheroidene spans the transmembrane region in the interhelical space of each LH1 αβ-heterodimer and is inclined at approximately 40° to the membrane plane and interacts mainly via hydrophobic forces with the αβ-heterodimer [α_(*n*)_-Met^34^ and β_(*n*)_-Val^22^], adjacent α_(*n*+1)_ apoprotein near its C terminus (Leu^36^, Val^29^, and Phe^25^), and the α_*n* − 1_ apoprotein at its N terminus (Phe^4^ and Ile^7^) (Fig. 4C and fig. S14). In addition, it interacts with the B880 BChl dimer within the LH1 αβ-heterodimer and one B880 in a neighboring αβ-heterodimer (Fig. 3D and fig. S14). The interactions of each carotenoid with *n* + 1, *n*, and *n* − 1 subunits and with bound BChls ensure the stabilization of carotenoids within LH1, excitation energy transfer from carotenoids to BChls, and effective cross-linking of the LH1 αβ-subunits.

Cofactors in the RC include four B800 BChls *a*, three BPhes, one 15-*cis*-carotenoid, one Fe^3+^ ion, and six UQ-10 molecules (Fig. 4D). The BChls of the RC are aligned on the same level as the LH1 B880 ring in the transmembrane region and have relatively equal distance with the closest LH1 BChls (Fig. 4B and fig. S13), providing the foundation for efficient energy transfer from LH1 to the RC. Unlike the RCs in other purple bacteria that have two BPhes (*6*–*9*), the cryo-EM structure reveals that the *Rba. veldkampii* RC L and M subunits accommodate three BPhes, which was confirmed by pigment extraction and high-performance liquid chromatography (HPLC) quantification analysis using the *Rhodospirillum* (*Rsp.*) *rubrum* RC-LH1 complexes as a reference (fig. S15; see Materials and Methods). The extra BPhe was assigned to a well-resolved planar density toward the outside of the M subunit with its center seemingly coordinated by a nearby peptide bond oxygen (fig. S16).

The cryo-EM map also shows three potential densities of lipid molecules between the RC and LH1 subunits. The most abundant lipids in *Rba. veldkampii* membranes are phosphatidylethanolamines (PEs) and phosphatidylglycerols (PGs), whereas glycolipids, phosphatidylcholines (PCs), and diphosphatidylglycerols, including cardiolipins, are absent (*25*). Therefore, we tentatively assigned these lipid densities to PE with C18:0 side chains (Fig. 4, A and C) (*32*). These putative lipid molecules have similar locations as those observed in the *Tch. tepidum* RC-LH1 complex (*7*) and form close contacts with the neighboring residues in the RC and LH1 (fig. S17 and table S6). Phe^56^ of the RC-H subunit may form interactions with both LH1 (α-Arg^15^) and lipids.

### Multiple pathways of quinone/quinol exchange

**Fig. 5:**
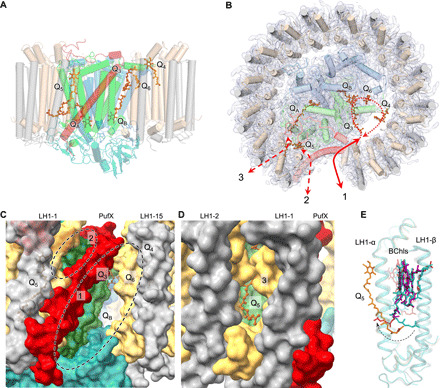
Channels of quinone/quinol exchange in the RC-LH1-PufX complex. (**A**) Distribution of six quinones in the RC-LH1-PufX complex (side view). (**B**) Periplasmic view of the locations of quinones in the RC-LH1-PufX complex from the periplasmic side and three potential pathways for quinone-quinol diffusion in the RC-LH1-PufX complex. The full arrow indicates the quinone pathway through the large opening in the LH1 ring created by PufX; dashed arrows represent the potential quinone-exchange routes through the Q_3_, Q_B_, Q_6_, and Q_4_ positions and through the relatively tighter channels between the LH1 subunits and between PufX and LH1. (**C**) Closer view of the gaps (circled) in the LH1 ring created by PufX, representing the pathways for quinone-quinol diffusion [pathways 1 and 2 as indicated in (B)]. Q_B_, Q_3_, Q_4_, and Q_6_ can be visualized through the larger gap (circle 1) between PufX and LH1-15. (**D**) The quinone-exchange channel between the LH1-1αβ and LH1-2αβ heterodimers [pathway 3 as indicated in (B)]. (**E**) Superimposed structures of LH1 αβ-heterodimers (first and second versus third and fourth) reveal explicitly the conformational change of the BChl tail near Q_5_, from outward facing to inward facing, which may play an important role in quinone transport within LH1. The color scheme of the first and second LH1-αβ pairs is the same as depicted in Fig. 1. The third and fourth LH1-αβ pairs for comparison are shown in teal.

Six molecules of UQ-10 were identified in the density map (Figs. 4, C and D, and 5A). Two UQ-10 molecules function as the primary (Q_A_) and secondary (Q_B_) quinone acceptors, with similar organizations to those of *Tch. tepidum* (fig. S18) (*7*). The head of Q_A_ is hydrogen bonded to His^220^ and Ala^261^ residues of the M subunit, and the head of Q_B_ is hydrogen bonded to the L-subunit residues His^191^ and Val^225^. Three additional putative UQ-10 molecules (Q_3_, Q_4_, and Q_6_) are located in the gap between LH1 and the RC and are mainly surrounded by nonpolar residues of the L and M subunits (Fig. 5B and fig. S18). The head of Q_3_ forms hydrogen bond with the L subunit Ser^179^. Q_3_ and Q_B_ have the same orientation and Q_3_ is in close proximity to the isoprenoid tail of Q_B_ (Figs. 4D and 5A and fig. S18), suggesting that Q_3_ is in a position appropriate for the exchange of Q_B_ after double reduction and protonation. A quinone molecule has also been identified at a similar position in the RC-LH1 complex of *Tch. tepidum* (fig. S22) (*7*).

Compared to a closed RC-LH1 monomer, an open RC-LH1 monomer exhibited increased diffusion rates of quinol and photosynthetic efficiency (*33*, *34*). The gap between the 1st and 15th LH1 αβ-heterodimers in the LH1 ring from *Rba. veldkampii* created by PufX (30 Å in distance) is the largest among those reported for purple bacterial RC-LH1 complexes (Fig. 5B), including the small gaps in the LH1 rings of *Rps. palustris* interrupted by protein W (*11*), of *Blc. viridis* by the missing γ-subunit (*9*), and of *Rfl. castenholzii* by the transmembrane helices of Cyt *c* and subunit X (*13*). These gaps have relatively similar locations within the LH1 ring (fig. S19 and movie S1), suggesting a specific channel for quinol/quinone exchange. The large space between PufX and the 15th LH1 αβ-subunits close to the cytoplasmic surface represents a channel for quinol diffusion, facilitating the transfer of reduced ubiquinone from the RC into the quinone pool outside the LH1 complex (Fig. 5C, circle 1). The space between PufX and the first LH1 αβ-subunits close to the periplasmic surface could be another route for quinone diffusion (Fig. 5C, circle 2, and fig. S20). Consistently, spectroscopic analysis has shown that PufX could facilitate the quinone-mediated redox interaction between the RC and Cyt *bc*_1_ in *Rba. veldkampii* and *Rba. sphaeroides* (*21*).

Molecular dynamics (MD) simulations on a 1-μs time scale reveal that Q_3_ moved toward the Q_B_ site and had a similar orientation as the Q_B_ quinone. Meanwhile, the Q_B_ quinone left the L subunit of the RC and moved toward the positions of Q_6_ and Q_4_ (fig. S21 and movie S2). In addition, Q_4_ displayed a remarkable movement in the lipid region between the RC and LH1 toward the PufX-formed large gap in the LH1 ring (fig. S21 and movie S3). The results suggest a potential quinone diffusion route: A quinone molecule enters the RC-LH1 complex through the large gap formed by PufX and accesses the position of Q_3_; the Q_3_ quinone replaces Q_B_, and the reduced Q_B_ moves to the Q_6_ and Q_4_ sites and subsequently diffuses out of the complex through the large opening in the LH1 ring (Fig. 5B).

Moreover, the isoprenoid tail of Q_5_ was found to be inserted into a space between the first and second LH1 αβ-heterodimers (Fig. 5D and fig. S20), representing a quinone in transit through a putative shuttling channel between the LH1 subunits. This channel is surrounded by hydrophobic residues of LH1 subunits and was also found in the closed LH1 rings of the RC-LH1 complexes from *Tch. tepidum* (*6*, *7*) and likely in the LH1 ring of *Rps. palustris* (*12*). Computational simulations on a modeled LH1 structure also suggested the possibility that ubiquinone can diffuse through the closed LH1 ring (*35*). In addition, the cryo-EM structure identified a conformational alternation at the phytol tail of the BChl close to Q_5_ (inward facing) in contrast to the tails of other LH1 BChls (outward facing) (Fig. 5E and fig. S22), presumably critical for generating a specific environment within the LH1 ring to facilitate the diffusion of Q_5_. Similar conformational variation of the phytol tails of BChls occurred also in the *Tch. tepidum* RC-LH1 structure (PDB ID: 5Y5S) (*7*), in which three BChls have inward-facing tails while those of the remaining LH1 BChls face outward (fig. S22).

Overall, the cryo-EM structure of the *Rba. veldkampii* RC-LH1-PufX supercomplex reveals the unique organization of PufX and the LH1 ring architecture with a large opening. This provides the structural basis for forming a stable photosynthetic core complex and ensures efficient proton transfer and quinone exchange across the LH1 ring through multiple pathways (Fig. 5B) necessary for anoxygenic photosynthesis and photosynthetic competence in the changing environment. The new structural model also highlights the natural variations of the photosynthetic RC-LH1 architectures.

## MATERIALS AND METHODS

### Protein purification

Wild-type *Rba. veldkampii* DSM-11550 [Deutsche Sammlung von Mikroorganismen und Zellkulturen (DSMZ), Germany] cells were grown under 1120 lumen light at 30°C for 7 to 14 days in flat glass bottles filled with anoxic sodium succinate 27 (N medium, DSMZ, Germany) medium to the top and tightly closed with magnetic stirring of 150 rpm (*21*). Cells were harvested by centrifugation at 5000*g* for 10 min. After washing twice with tris-HCl buffer (pH 8.0), cells were resuspended in the working buffer (20 mM HEPES-Na, pH 8.0) and disrupted by passage through a French press three times at 16,000 psi. Unbroken materials were removed by centrifugation at 20,000*g* for 30 min. Membranes were collected by centrifugation at 125,000*g* for 90 min and solubilized by 3% (w/v) *n*-dodecyl β-d-maltoside (DDM) for 15 min in the dark at 4°C with gentle stirring. After the unsolubilized materials were removed by centrifugation at 21,000*g* for 30 min, the clarified supernatant was applied onto the 10 to 25% (w/v) continuous sucrose gradient made with the working buffer containing 0.01% (w/v) DDM. Gradients were centrifuged at 230,000g for 19 hours. The core complex, represented by the heaviest red/brown pigmented band, was collected and further purified by a Sephacryl S200 gel filtration column (Cytiva). The pooled fractions used for cryo-EM data collection show an absorbance ratio at 871/805 nm, which represents the absorbance ratio of RC-LH1/LH2 of 2.84.

### Cryo-EM data collection

Three microliters of the purified RC-LH-PufX complex was applied to the glow-discharged holey copper grids (Quantifoil Cu R1.2/1.3, 300 mesh) with a thin carbon-supported film. The grid was plunge-frozen in liquid ethane using a Vitrobot Mark IV (Thermo Fisher Scientific). Parameters for plunge-freezing were set as follows: blotting time, 3 s; waiting time, 30 s; blotting force, 0; humidity, 100%; and chamber temperature, 4°C. Data were collected at the University of Tokyo on a 300-kV Titan Krios electron microscope (Thermo Fisher Scientific) with a K3 direct electron detector (Gatan) in counting mode. A total of 5022 movies were recorded at nominal magnification of ×105,000 and a pixel size of 0.83 Å/pixel, with a defocus range between 0.8 and 1.8 μm and a dose rate of 1.0 electrons per Å^2^ per frame. A typical motion-corrected cryo-EM image is shown in fig. S1A.

### Data processing

The movie stacks were motion corrected using MotionCor2 (*36*). Contrast transfer function (CTF) and defocus values were estimated by CTFFIND-4.1 (*37*). A total of 1,168,396 particles were automatically picked using crYOLO (*38*) with the input box size of 250 × 250 pixels. Particles were then extracted in a box size of 64 pixels in a 3.24-Å/pixel size using RELION 3.1 (*39*, *40*) to speed up initial dataset cleanup consisting of two subsequent reference-free 2D classifications followed by three rounds of 3D classifications, for which a 3D initial model was calculated in RELION 3.1. During each classification step, the particles that were categorized into poorly defined classes were rejected. The remaining 644,294 particles were reextracted in a 0.83-Å/pixel size. After another initial 3D model calculation and the subsequent four rounds of 3D classification, the resulting 184,921 good particles were refined at 3.7-Å resolution on the basis of Fourier shell correction (FSC) 0.143 criterion in RELION 3.1. Per-particle CTF refinement and beam tilt estimation were performed. Bayesian polishing and an additional round of 3D auto-refinement generated the final maps at 2.84-Å resolution on the basis of the FSC 0.143 criterion. Local resolution was estimated in RELION 3.1.

### Model building and refinement

For the RC, the crystal structure of *Rba. sphaeroides* RC (PDB ID: 1M3X) (*41*) was fitted to the cryo-EM map as a rigid body using “fit in map” in Chimera (*42*). For the LH1 ring, a single pair of LH1 from the crystal structure of *Tch. tepidum* RC-LH complex (*7*) was fitted to the cryo-EM map. The RC and LH1 structures were then manually adjusted using Coot (*43*). Then, 30 BChls (PDB ligand ID: BCL) of LH1, 15 spheroidene pigments (14 belonging to LH1 and 1 to RC, PDB ligand ID: SPO), and 4 UQ-10 molecules (PDB ligand ID: U10) outside of the RC were fitted to the cryo-EM map. PufX was built manually using Coot.

*Rba. veldkampii* and *Rba. sphaeroides* differ in their polar lipids. The most abundant lipids in *Rba. veldkampii* membranes were previously found to be PE, PG, and an unidentified lipid, whereas glycolipids, PCs, and diphosphatidylglycerols, including cardiolipins, were absent in *Rba. veldkampii* (*25*). Therefore, we assigned the potential lipid densities to the major and mostly fitted lipid PE in the *Rba. veldkampii* RC-LH1-PufX structural model.

The final model was refined by Phenix (*44*), and the stereochemistry was assessed by MolProbity (*45*). Statistics for cryo-EM data collection and model refinement are summarized in table S1. Amino acid sequences of protein polypeptides in the RC-LH1-PufX complex from *Rba. veldkampii* are shown in fig. S23. Images were generated and analyzed by UCSF Chimera. The mapping of electrostatic potential was achieved using PyMOL with the Adaptive Poisson-Boltzmann Solver (APBS) Electrostatics plugin (https://pymolwiki.org/index.php/APBS_Electrostatics_Plugin). The potential tunnels for quinone diffusion were calculated by Hollow 1.3 (*46*) and the channel volumes were calculated by 3V server (http://3vee.molmovdb.org/volumeCalc.php).

### Pigment extraction from RC-LH1 complexes

The RC-LH1 complexes from *Rba. veldkampii* and *Rsp. rubrum* were isolated from sucrose density gradients as described above. The samples were concentrated in an Amicon Ultra 15-ml centrifugal filter with a 100-kDa membrane at 3000*g*. Pigments were extracted from concentrated complexes by the addition of 5 volumes of 7:2 (v/v) acetone:methanol and incubated on ice in the dark for 10 min. Precipitated proteins were separated from the extracted pigments by centrifugation at 14,000*g* for 10 min, and the solvent was further clarified by passage through a 0.22-μm hydrophobic polytetrafluoroethylene (PTFE) syringe filter. Pigments were then immediately analyzed by reversed-phase HPLC.

### Analysis and quantification of pigments

BChls and BPhes were separated at 1 ml·min^−1^ at 40°C on a Supelco Discovery HS C18 (5-μm particle size, 120-Å pore size, 250 × 4.6 mm) on an Agilent 1100 HPLC system using a program modified from a previously published method (*47*). Solvents A and B were 64:16:20 (v/v/v) methanol:acetone:H_2_O and 80:20 (v/v) methanol:acetone, respectively. Pigments were eluted at 50% solvent B held for 2 min, followed by a linear gradient to 100% solvent B over 10 min, and held at 100% solvent B for 25 min. Elution of BChl and BPhe was monitored by checking the absorbance at 747 nm. Peak areas for BChl and BPhe in each complex were calculated using the integration function in Agilent Chemstation software. BChl peak areas from each chromatogram were normalized, and relative BPhe peak areas were used to calculate the BPhe content of the *Rba. veldkampii* RC-LH1 complex based on the known ratio of BChl:BPhe in the RC-LH1 complexes of *Rsp. rubrum* (36:2) (*48*) as a reference. Three independent biological replicates were measured and analyzed.

### MD simulation

The initial structure for MD simulation was prepared as follows: The structures of hydrocarbon chains of UQ-10 and BPhe (BPH), which are missing in the cryo-EM structure, were modeled manually. The topologies and the force field parameters of BPH, spheroidene (SPO), and UQ-10 were generated using the “Ligand Reader & Modeler” function (*49*) of the CHARMM-GUI server (*50*) and the CHARMM general force field (CGenFF) (*51*). The topology of BChl *a* (BCL) was generated by replacing the two protons of the bacteriochlorin moiety of BPH with a Mg^2+^ ion. The charge of the Mg^2+^ ion was the sum of the charges of the two protons. The rotational and translational position of the protein complex in a lipid bilayer was determined using the Positioning of Proteins in Membrane (PPM) server (*52*). The protein complex was embedded in a solvated lipid bilayer consisting of 1-palmitoyl-2-oleoyl-*sn*-glycero-3-phosphoethanolamine (POPE) molecules using the “Membrane Builder” function (*53*) of the CHARMM-GUI server. The system was composed of 34 protein chains, 34 BCL, 15 SPO, 3 1,2-distearoyl-*sn*-glycero-3-phosphoethanolamine, 3 BPH, 6 U10, and 551 POPE; an Fe^3+^, 179 K^+^, and 183 Cl^−^ ions; and 65,692 water molecules. The total number of the atoms was 312,747, and the size of the initial system was 16.0 nm × 16.0 nm × 12.9 nm. Transferable Intermolecular Potential 3-Point (TIP3P) model (*54*) was used for the water molecules. The CHARMM36m force field (*55*) was used for the protein chains, and the CHARMM36 force field (*56*, *57*) was used for the other molecules. Distance restraints with the force constant of 1.0 × 10^5^ kJ∙nm^−2^ were imposed between the metal ions Mg^2+^ and Fe^3+^ and their coordinating atoms. After the system was energy minimized and equilibrated, 1-μs MD simulations were performed twice with different initial velocities. During the MD simulations, the temperature was kept at 303.15 K using the Nosé-Hoover method (*58*), and the pressure was kept at 1.0 × 10^5^ Pa using the Parrinello-Rahman method (*59*, *60*). Bond lengths involving hydrogen atoms were constrained using the Linear Constraint Solver (LINCS) algorithm (*61*, *62*) to allow the use of a large time step (2 fs). Electrostatic interactions were calculated with the particle mesh Ewald method (*63*, *64*). All MD simulations were performed with GROMACS 2020 (*65*), with coordinates recorded every 10 ps.

## SUPPLEMENTARY MATERIALS

Supplementary material for this article is available at http://advances.sciencemag.org/cgi/content/full/7/25/eabf8864/DC1

## Appendix

View/request a protocol for this paper from *Bio-protocol*.
